# Cation complexation by mucoid *Pseudomonas aeruginosa* extracellular polysaccharide

**DOI:** 10.1371/journal.pone.0257026

**Published:** 2021-09-02

**Authors:** Oliver J. Hills, James Smith, Andrew J. Scott, Deirdre A. Devine, Helen F. Chappell

**Affiliations:** 1 School of Food Science & Nutrition, University of Leeds, Leeds, United Kingdom; 2 School of Chemical & Process Engineering, University of Leeds, Leeds, United Kingdom; 3 School of Dentistry, University of Leeds, Leeds, United Kingdom; Indian Institute of Technology Kharagpur, INDIA

## Abstract

Mucoid *Pseudomonas aeruginosa* is a prevalent cystic fibrosis (CF) lung colonizer, producing an extracellular matrix (ECM) composed predominantly of the extracellular polysaccharide (EPS) alginate. The ECM limits antimicrobial penetration and, consequently, CF sufferers are prone to chronic mucoid *P*. *aeruginosa* lung infections. Interactions between cations with elevated concentrations in the CF lung and the anionic EPS, enhance the structural rigidity of the biofilm and exacerbates virulence. In this work, two large mucoid *P*. *aeruginosa* EPS models, based on *β*-D-mannuronate (M) and *β*-D-mannuronate-*α*-L-guluronate systems (M-G), and encompassing thermodynamically stable acetylation configurations–a structural motif unique to mucoid *P*. *aeruginosa*–were created. Using highly accurate first principles calculations, stable coordination environments adopted by the cations have been identified and thermodynamic stability quantified. These models show the weak cross-linking capability of Na^+^ and Mg^2+^ ions relative to Ca^2+^ ions and indicate a preference for cation binding within M-G blocks due to the smaller torsional rearrangements needed to reveal stable binding sites. The geometry of the chelation site influences the stability of the resulting complexes more than electrostatic interactions, and the results show nuanced chemical insight into previous experimental observations.

## Introduction

Bacterial biofilms consist of a community of bacteria embedded in an extracellular matrix (ECM) of polysaccharide. More widely dispersed at longer length scales, are polypeptide oligomers and extracellular proteins, and circular polynucleotides such as plasmids [1]. The ECM limits the penetration of antimicrobials, which contributes to the minimum inhibitory concentrations of antimicrobials against biofilms being 100-1000-fold higher than those required for treating planktonic bacteria [2]. *Pseudomonas aeruginosa* is one such species where its biofilm is definitively associated with chronic disease, most notably in the cystic fibrosis lung [3]. Confocal laser scanning microscopy and fluorescent lectin-binding analysis has characterized *P*. *aeruginosa* biofilm matrix architecture *in situ* and shown that the bacterial microcolonies are embedded in an open 3D network of matrix material [4,5]. This network gives rise to interstitial void spaces, to which, the vast majority of biofilm (bulk) water is confined [6].

The cystic fibrosis (CF) lung acts as a prime infection site for *P*. *aeruginosa* [7] and quantitative microbiological analysis of CF sputum over long periods of time has demonstrated that it is the most prevalent, and most dangerous, pathogen found in CF patients [8]. Initial colonization is by the non-mucoid phenotype but over time the stress of the CF lung environment affects the conversion to the mucoid phenotype, which becomes the dominant variant [9]. *P*. *aeruginosa* is intrinsically resistant to antibiotic therapy due to its low outer membrane permeability, production of antibiotic inactivating enzymes and expression of efflux pumps [10]. The mucoid biofilm ECM further adds to its pathogenicity and mature biofilms are rarely eradicated by high doses of antimicrobial treatments such as tobramycin [11]. Consequently, chronic infection by mucoid *P*. *aeruginosa* leads to decreased lung function and, ultimately, death [12].

The polysaccharide component of the mucoid *P*. *aeruginosa* ECM is predominantly composed of the unbranched anionic polysaccharide alginate, a copolymer of two uronate sugars, namely, *β*-D-mannuronate (M) and its C5 epimer *α*-L-guluronate (G), linked via a 1–4 glycosidic bond [13,14]. The alginate polysaccharide is acetylated at the C2 and/or C3 position(s) exclusively on the M units, inferred from the absence of H^1^-NMR chemical shifts characteristic of acetylated G-units of bacterial alginates [15]. Bacterial alginate H^1^-NMR spectra also show the parallel presence of both mono-acetylated and diacetylated M-units [16], which suggests that steric bulk at one carbon position following acetylation does not prevent acetylation at the other carbon position. Moreover, H^1^-NMR spectroscopy has quantified the degree of M-unit acetylation in bacterial alginates to be between 4–57% [16]. Acetylation offers protection against epimerase activity [17] and consequently repetitions of G units (G-blocks), which are the distinguishing architecture of algal alginate, do not occur in bacterial alginate [18].

Despite the fact that biofilm matrices are highly solvated systems, the ECM has the physical characteristics of a solid (viscoelastic) material, evident from the high storage modulus and low loss modulus [19]. It adopts a gel-like structure, whereby the internal polymer network is stabilized by chemical cross-links including electrostatic interactions, hydrogen bonds and dispersive interactions [19]. Binding of amphiphilic fluorescent carbon dots to the *P*. *aeruginosa* ECM has shown, in the absence of cations, the ECM is dendric in morphology, stabilized solely through entanglements [20].

In the CF-lung, as in all biological tissues, the most common serum metal ion is sodium. A recent study suggested that salinity levels, and thus sodium ion concentration, in CF-lung sputum were slightly higher than in control subjects, although statistical significance was only achieved in age-matched populations [21]. When considering other metal ions, samples of expectorated sputum from cystic fibrosis sufferers and a non-CF control group, have shown significantly (*p* <0.001) elevated levels of magnesium (30 mg/L), calcium (102 mg/L), iron (797 μg/L) and zinc (1285 μg/L) [21]. Apart from iron, which is also associated with bacterial virulence and respiration [22], these ions are known to be implicated in a variety of inflammatory pathways [23,24]. Across all CF patients iron, magnesium and zinc had the largest increase compared to non-CF sputum samples [21]. However, when looking specifically at samples from patients with *P*. *aeruginosa* infections, it was only magnesium (*p*<0.05) and calcium (*p*<0.01) that showed significant elevation compared to samples from patients with other common CF-infections [21]. Furthermore, there was a high correlation coefficient between calcium and magnesium in the CF sputum samples [21].

These ions serve to create permanent electrostatic cross-links in the extracellular polysaccharide (EPS), established between neighbouring M-M and M-G junctions [25], further stabilizing the ECM [26]. Using compression measurements on mucoid biofilms, calcium ions have been observed to be strong cross-linkers, enhancing structural rigidity through an increased Young’s modulus [27]. Moreover, the presence of calcium ions increases the amount of alginate produced leading to thicker, more granular, biofilm structures [28]. Indeed, experiments conducted in continuous-flow stirred tank reactors, show that calcium ions create a biofilm where specific cellular growth outstrips the specific cellular detachment rate, leading to biofilm thickening and accumulation [29]. Relative to calcium ions, sodium ions appear to be poor cross-linkers, forming weaker, temporary cross-links and softer gel-like structures stabilized though entanglements rather than bridging cations [30,31]. NMR studies on ^13^C-labelled native *P*. *aeruginosa* biofilms showed that while calcium ions caused broadening of the CHOH-carbon signals (particularly within the guluronate units) as gel formation progressed and molecular mobility reduced, the effect of magnesium in the same system was significantly less pronounced [25].

Cation binding by algal alginate has been studied theoretically, using classical molecular dynamics (MD) and quantum chemical Density-Functional Theory (DFT). A combination of MD and Monte Carlo simulations, studying calcium ion complexation by two poly-*α*-L-guluronate (G) chains, suggested that chain complexation is facilitated through ionic interactions between the cations and carboxylate groups [32,33]. Upon establishment of calcium-carboxylate ionic interactions, driving initial poly-*α* -L-guluronate aggregation, a hydrogen-bonding network is then established between chains, although the stability of the resulting aggregate is more dependent on the calcium cross-links [34]. By contrast, MD simulations of sodium ions with poly-*α* -L-guluronate indicates that no bonding interaction occurs as the monovalent ion sits too deeply in the G-G junctions and is, therefore, too distant to attract a second chain [35]. Similarly, a lack of sodium-induced bonding is also observed in analogous poly-*β*-D-mannuronate simulations [36].

More accurate DFT calculations, investigating complexation of divalent cations (Mg, Ca, Sr, Mn, Co, Cu, Zn) by simple algal disaccharide polyuronates (M-M, G-G and M-G junctions) highlight the role of the hydroxyl, glycosidic and ring oxygen atoms in cation binding. It was shown that in these systems the alkaline earth cations in particular, can, in principle, form five or six ionic bonds within each complex [37,38]. In these circumstances, the cation-carboxylate bonds are stronger relative to the hydroxyl, glycosidic and ring oxygen-cation bonds, as indicated by their shorter bond lengths. A more recent study examining the structure and reactivity of the M-M, G-G, M-G and G-M conformations, showed that the stability, as defined by the hardness (η), of the cation-disaccharide complexes decreased from magnesium to calcium to sodium, a trend inversely proportional to the ionic radius [39]. This suggests that magnesium-cross-linked alginate chains are more stable than those cross-linked with calcium. However, C^13^-NMR spectroscopy measurements of the interaction of *P*. *aeruginosa* alginate, with bivalent metal ions does not corroborate this result, suggesting instead that binding of magnesium ions to the bacterial alginate framework is weak and non-specific [25].

In summary, previous molecular modelling studies have assessed the contributions specific cations make to the stability of algal alginate complexes upon the establishment of cross-links, but there remains disagreement as to which ions produce the most stable structures, and what the chemistry of the interactions between those cations and the alginate looks like. Attention has been drawn to the importance of the cation-carboxylate interaction during the aggregation events and although coordination to other oxygen functionality is possible, this has not been considered vital for aggregation. In the present work, the interaction between cations that are elevated in the CF lung, and the extracellular polysaccharide, was studied with aim of quantifying how and where the cations contribute to EPS stability. Two simplified mucoid *P*. *aeruginosa* EPS molecular models were constructed that possess structural motifs unique to mucoid *Pseudomonas*. Each model consisted of two chains, each containing four saccharide units. DFT studies followed, to understand the chemical interactions between the chains and selected cations, to determine the stable coordination geometries and to quantify the thermodynamic stability of the resulting complexes. The choice of metal ions in this study was based on the highest concentrations in CF sputum, specifically, the three metal ions (Na^+^, Ca^2+^, Mg^2+^), with the sodium ion effectively acting as a biological control [21]. Compared to non-CF controls, the calcium ion concentration was 7.5 × higher and the magnesium ion concentration 2.5 × higher [21]. The models predict the potent cross-linking ability of the ions relative to one another and indicate that stable cation complexation results from a combination of electrostatic and steric factors.

## Materials & methods

### Computational details

All geometry optimizations were performed using the plane-wave Density Functional Theory (DFT) code, CASTEP [40]. For all polyuronate and ion-complexation optimizations, a convergence tested cut-off energy of 900 eV was employed, as well as a Monkhorst-Pack *k*-point grid of 1 x 1 x 1 to sample the Brillouin zone [41] in an orthorhombic box of size 45 Å x 27 Å x 16 Å. On-the-fly ultrasoft pseudopotentials were used [42] alongside the PBE exchange-correlation functional [43]. The semi-empirical dispersion correction of Tkatchenko and Scheffler [44] was employed to account for intra- and intermolecular dispersive forces. The SCF tolerance was set to 1×10^−7^ eV Atom^-1^ and the energy, force and displacement tolerances for the geometry. optimisations were set to 1×10^−5^ eV Atom^−1^, 0.03 eV Å^−1^ and 1×10^−3^ Å respectively. Following each geometry optimization, Mulliken bond populations [45] were calculated to classify the nature of bonding in each of the complexed structures. All molecules were generated and visualized using *CrystalMaker*^*®*^ [46]. For the determination of formation energies, chemical potentials for sodium, magnesium and calcium were calculated by their respective 0K energy per atom, from the pure metals in their lowest energy configurations, namely HCP (sodium), hexagonal (magnesium) and BCC (calcium). Chemical potentials for hydrogen and oxygen were calculated from optimized single molecules, as was the energy of the ethanal molecule. All calculations were conducted at the same cut-off energy of 900 eV.

### Molecular models

Mucoid *P*. *aeruginosa* alginate presents *in vivo* with acetylated M units and no contiguous G-blocks [15,18]. This creates structural variations throughout the EPS architecture as certain fractions of the EPS are exclusively mannuronate while in others, guluronate units are successfully incorporated. As such, two molecular templates were created. The first was a poly-*β*-D-mannuronate structure, a single chain of four M units linked via a 1–4 glycosidic bond. The second was a copolymeric *β* -D-mannuronate-*α*-L-guluronate structure, a single chain of two M units and two G units linked via a 1–4 glycosidic bond in an alternating M-G pattern.

Fifty percent acetylation of these templates at the C2 or C3 positions on the M units was then performed. This degree of acetylation falls within the 4–57% range that has been observed experimentally [15]. For the poly-*β*-D-mannuronate and copolymeric *β* -D-mannuronate-*α*-L-guluronate structures, this gave 12 and 4 possible acetylation configurations respectively. The thermodynamic stability of each acetylation configuration (Table 1) was determined by means of evaluating a formation energy (*E*_*f*_) according to Eq 1 where *E*_*final*_ is the final energy of the acetylated structure, *E*_*initial*_ is the energy of the initial non-acetylated template, *E*_*ethanal*_ is the energy of an ethanal molecule and *μ*_*H*_ is the chemical potential of a hydrogen atom. For the poly-*β* -D-mannuronate structure, *n = 2* and *m = 4* and for the copolymeric *β* -D-mannuronate- *α* -L-guluronate structure, *n = 1* and *m = 2*.


$$E_{f}=E_{final}-(E_{initial}+{nE}_{ethanal}-{m\mu }_{H})$$ (1)

**Table 1:** Formation energies (eV) of the different acetylation configurations, using nomenclature outlined in Fig 1, for each polyuronate template.

*β*-D-mannuronate	
Acetylation configurations	*E*_*f*_(*eV*)
M1(C2) M2(C2)	-0.10
M1(C2) M2(C3)	+0.20
M1(C3) M2(C2)	-0.18
M1(C3) M2(C3)	-0.16
M1(C2) M3(C2)	+0.36
M1(C2) M3(C3)	-0.14
M1(C3) M3(C2)	-0.24
M1(C3) M3(C3)	+0.46
M1(C2) M4(C2)	+0.09
M1(C2) M4(C3)	+0.50
M1(C3) M4(C2)	+0.01
M1(C3) M4(C3)	+0.17
*β*-D-mannuronate-*α*-L-guluronate	
Acetylation configurations	*E*_*f*_(*eV*)
M1(C2)	+0.06
M1(C3)	+0.11
M2(C2)	+0.29
M2(C3)	-0.06

**Fig 1:**
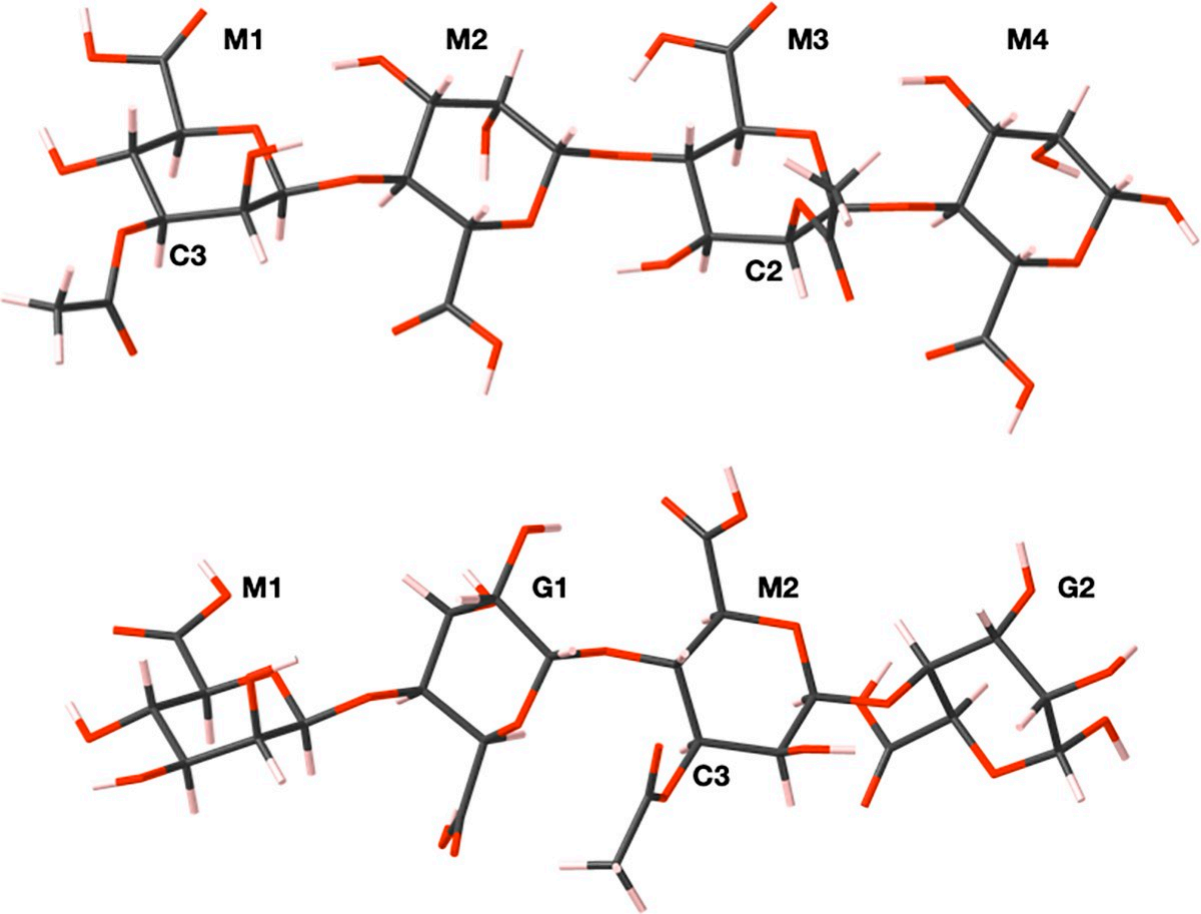
Examples of thermodynamically stable (top) acetylated poly-*β*-D-mannuronate (PolyM) configuration and (bottom) the copolymeric *β*-D-mannuronate-*α*-L-guluronate (PolyMG) configurations. Carbon atoms are shown in grey, oxygen in red and hydrogen in pink. The uronate units and acetylated carbon atom positions are labelled for clarity.

The most thermodynamically stable acetylated poly-*β*-D-mannuronate and copolymeric *β*-D-mannuronate-*α*-L-guluronate structures are shown in Fig 1 and from here on are referred to as PolyM and PolyMG respectively.

### Water

Pulsed-field gradient NMR has shown that biofilm bulk water is highly mobile and confined to channels within the *P*. *aeruginosa* biofilm matrix. A small amount of water is present entrapped within the secondary structures of polysaccharides but is exchanged frequently with the bulk solvent [6]. Additional NMR observations on water movement through polysaccharide gels provide consistent conclusions, highlighting that waters near to the polysaccharide do not have a significantly reduced motion, i.e. the water does not bind specifically [47]. Binding of ions by polysaccharides occurs preferentially in positions where oxygen functionality is well-positioned to displace water from the coordination shell of the cation,[48] a requirement satisfied by positioning ions in-between chains. For these reasons, as well as for reduced computational expense, all optimisations were performed *in vacuo* with the omission of water molecules. This allowed for large scale, tractable, DFT calculations focusing solely on cation-polysaccharide interactions, which gave thermodynamic predictions in line with experimental observation.

## Results & discussion

### 2-chain systems

The most thermodynamically stable arrangement of two PolyM and PolyMG chains was evaluated. The configurations individually tested were parallel, antiparallel, parallel-inverted (where one chain has been inverted 180° about the chain axis) and antiparallel-inverted, with all atoms being given complete freedom. The thermodynamic stability of each arrangement was determined by evaluation of the formation energy according to Eq 2 where *E*_*final*_ is the final energy of the spatial arrangement and *E*_*initial*_ is the energy of a single PolyM or PolyMG structure.


$$E_{f}=E_{final}-{2E}_{initial}$$ (2)

The formation energies of all configurations are presented in Table 2.

**Table 2:** Formation energies (eV) of the different spatial arrangements of two PolyM and PolyMG chains.

Spatial arrangement	*E*_*f*_ (eV)	
	PolyM	PolyMG
Parallel	−2.27 (2)	−2.87 (6)
Antiparallel	−2.29 (4)	−1.90 (4)
Parallel-inverted	−0.93 (1)	−1.92 (3)
Antiparallel-inverted	−1.66 (2)	−2.65 (4)

The number of hydrogen bonds established between chains for each spatial arrangement is shown in parentheses.

**Fig 2:**
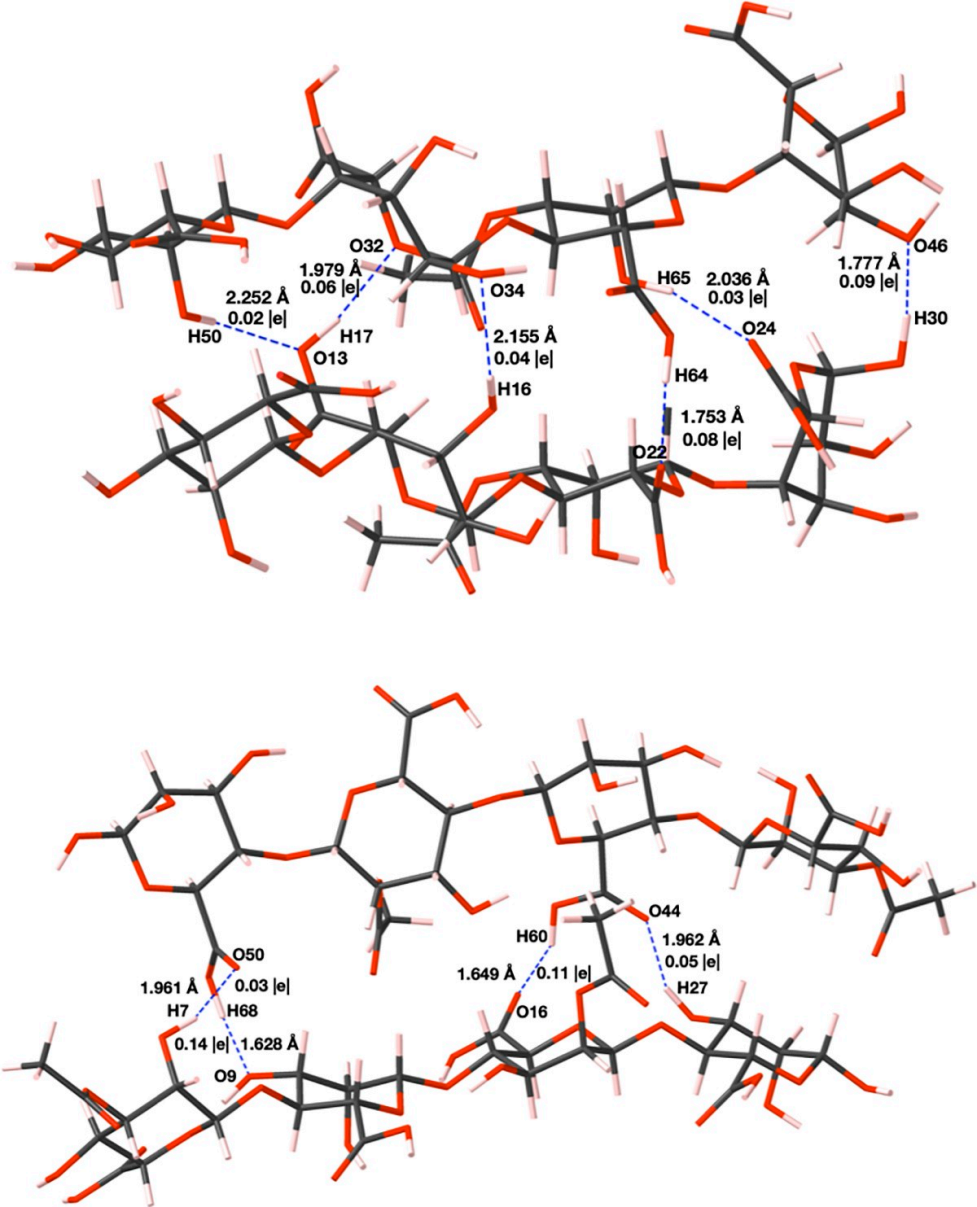
Antiparallel arrangement of two PolyM structures (PolyM_(ap)_, top) and parallel arrangement of two PolyMG structures (PolyMG_(P)_, bottom). Carbon atoms are shown grey, oxygen red and hydrogen pink. Hydrogen bonds between chains are shown as blue dashed lines and the Mulliken populations and lengths of these bonds are marked.

The antiparallel arrangement of two PolyM structures and the parallel arrangement of two PolyMG structures were the most thermodynamically stable spatial arrangements and are shown in Fig 2. The antiparallel polyM and parallel polyMG arrangements, compared to the other spatial arrangements tested, establish a larger hydrogen bonding network between chains. The network is larger in the parallel PolyMG system compared to the antiparallel PolyM system as, within M-G junctions, there is a wider variety of hydrogen-bonding functionality at a suitable orientation to sustain more hydrogen bonds. For example, oxygen functionality that participates in hydrogen-bonding is limited to carboxyl and hydroxyl groups in the antiparallel PolyM system but extends also to the glycosidic oxygen in the parallel PolyMG system. This helps rationalize the greater stability of the hydrogen bonded parallel PolyMG system. Hereafter, the antiparallel PolyM and parallel PolyMG arrangements are referred to as PolyM_(ap)_ and PolyMG_(p)_ respectively.

Dihedral angles (ϕ, ψ) around the glycosidic bonds for the PolyM and PolyMG molecular models are given in S1 Table. For the minimum energy PolyM_(ap)_ system, ϕ and ψ fall in the range of -54 to -107˚ and -123 to -156˚ respectively. For the PolyMG_(p)_ system these ranges are -47 to -101˚ and -55 to -144˚ respectively. These torsional angles match well with the most energetically favourable helical conformations of poly-*β*-D-mannuronate and poly-*α*-L-guluronate hexamers calculated by Braccini *et al* using a molecular mechanics method [49]. In the work of Bekri *et al*. [50] and Agulhon *et al*., [37] the two dihedral angles involved in the glycosidic linkage in M-M, M-G, G-M and G-G diuronates were calculated using DFT (B3LYP) and Hartree-Fock (HF) levels of theory, respectively. Both works identified multiple different minima corresponding to different values of (ϕ, ψ). B3LYP gave (ϕ, ψ) angles for minimum energy M-M, M-G, G-M and G-G diuronates of (312˚, 92˚), (57˚, 248˚), (269˚, 202˚) and (270˚, 203˚), respectively [50]. HF gave (ϕ, ψ) angles for minimum energy M-M and G-G diuronates of (274˚, 344˚) and (305˚, 292˚) respectively [37]. It is important to note that during the construction of the molecular models, in this work, all atoms were given complete freedom and our ground-state structures were not identified by constrained conformational searching. Moreover, the polyuronate molecular models are larger in molecular weight and possess acetyl functional groups, both of which effect the axial conformational flexibility of the polyuronate. It is unsurprising therefore that the (ϕ, ψ) angles obtained in this work differ from the those reported in both the works of Bekri *et al*. and Agulhon *et al*. Although it is clear that torsional space is a complex potential energy landscape, encompassing multiple local minima, that correspond to different dihedral angles (ϕ, ψ), it is nevertheless a useful measure to compare polyuronate configurations in this study. Both the PolyM and PolyMG systems possess oppositely displaced carboxyl groups, a feature also present in the minimum energy M-M and G-G diuronates in the work of Agulhon *et al*., [37] and, M-M and M-G junctions shifted to lower angles of *ϕ*, an observation replicated by Bekri *et al* [50].

### Thermodynamic stability of the cation cross-linked 2-chain systems

Geometry optimisations of single sodium, calcium and magnesium ions at multiple different starting points along the length of a single PolyM and PolyMG chain, in the vicinity of the M-M and M-G junctions, was carried out. The most favourable positions (S1–S3 Figs and S2 Table) demonstrated that in the stable binding positions each ion formed multiple oxygen contacts to hydroxyl, acetyl, ring, glycosidic and carboxylate oxygen atoms. This aligns with observations from experimental crystal structures of calcium-carbohydrate complexes, which show that stable binding positions adopted by calcium ions occur in regions where the ion can adopt multiple bonds to polysaccharide oxygen functionality [48]. Moreover, charge-saturated PolyM and PolyMG structures (i.e., binding to multiple cations), with respect to all four carboxylic acid groups, gave more thermodynamically stable structures compared to the single ion complexes. The greater stability of these charge saturated structures justifies the placing of multiple, rather than single, cations in-between the 2 chains.

Therefore, to study cation-extracellular polysaccharide interactions, of cations typically found in cystic fibrosis sputum, 8 Na^+^, 4 Mg^2+^ and 4 Ca^2+^ ions were positioned in-between chains in the PolyM_(ap)_ and PolyMG_(p)_ systems, in regions where multiple cation-oxygen contacts could be sustained and in the vicinity of carboxylate groups. These positions were determined by reference to the lowest energy binding positions observed in the single-chain studies above. Hydrogen atoms were removed from the carboxylic acid groups to ensure charge balance, and the number of cations included represented a fully charge-saturated system with respect to the carboxylic acid groups. Full geometry optimizations were performed and the thermodynamic stability was determined by means of evaluating a formation energy according to Eq 3 where *E*_*final*_ represents the final energy of the optimized complex, *E*_*initial*_ represents the energy of the PolyM_(ap)_ or PolyMG_(p)_ systems, *μ*_*A*_ is the chemical potential of the cation and *μ*_*H*_ is the chemical potential of a hydrogen atom.


$$E_{f}=E_{final}-(E_{initial}+{n\mu }_{A}-m\mu_{H})$$ (3)

The formation energies for the cation cross-linked PolyM_(ap)_ and PolyMG_(p)_ systems are given in Table 3.

**Table 3:** Formation energies (eV) for the cation cross-linked PolyM_(ap)_ and PolyMG_(p)_ complexes.

System	*E*_*f*_ (eV)		
	Na^+^	Mg^2+^	Ca^2+^
PolyM_(ap)_	+0.62	-4.27	-9.53
PolyMG_(p)_	-1.53	-5.95	-10.01

Across all sputum ions, more stable complexes are formed (by 0.5–1.5 eV) with PolyMG_(p)_, highlighting a slight preference for binding within M-G-blocks. This is consistent with elevated guluronic levels increasing metal ion affinity in algal alginates [51]. The 2-chain calcium complexes are very stable relative to the PolyM_(ap)_ and PolyMG_(p)_ systems without cations (Fig 2), as expected given experimental observations when the *P*. *aeruginosa* ECM is exposed to calcium [26]. For both polyuronate systems, the calcium ions produce more stable cross-linked structures relative to both sodium (~9 eV) and magnesium (~5 eV). At the atomistic level, this provides a thermodynamic rationale behind the increase in gelation capability of alginates upon substitution of extracellular sodium for calcium ions, which has been observed experimentally and through MD simulations [31,52]. However, it should be noted that it is in contradiction to a recent DFT study on disaccharides that predicted that magnesium would produce the most stable cross-linked structures [50]. This suggests that the larger model we are employing here better predicts the actual chemistry of the *in vitro* alginate structures.

From the formation energies in Table 3, it is clear that sodium ions establish the weakest cross-links between chains and it is thermodynamically unfavourable (with a positive formation energy) for the PolyM_(ap)_ system to aggregate about them. It can be interpreted that sodium ions are unable to induce the aggregation of bacterial alginate structures that only possess (acetylated) M-blocks. Indeed, even for *P*. *aeruginosa*, rheological experiments on its sodium alginate ECM, show that the chains are stabilized by entanglements and form only weakly held together, transient ionic networks [30]. Given the ubiquity of sodium in the extracellular environment, this result is not unexpected. Magnesium ions have a weaker interaction with the extracellular polysaccharide (Table 3) relative to calcium ions, which supports C^13^-NMR observations showing magnesium having a much smaller impact on the line broadening of the *P*. *aeruginosa* ECM chemical shifts compared to calcium, particularly at low concentrations [25]. For both polyuronate systems, the stability trend follows the order Ca^2+^ > Mg^2+^ > Na^+^. This is a trend that matches experimentally determined metal-alginate affinities [53], as well metal ion affinities demonstrated by acetylated bacterial polysaccharides in ion chromatography experiments [54]. However, this trend is not inversely proportional to the ionic radius (Ca^2+^ ~ Na^+^ > Mg^2+^), meaning the charge density of the ions is not the only factor that dictates cross-linked network stability.

### Cation coordination geometries

**Fig 3:**
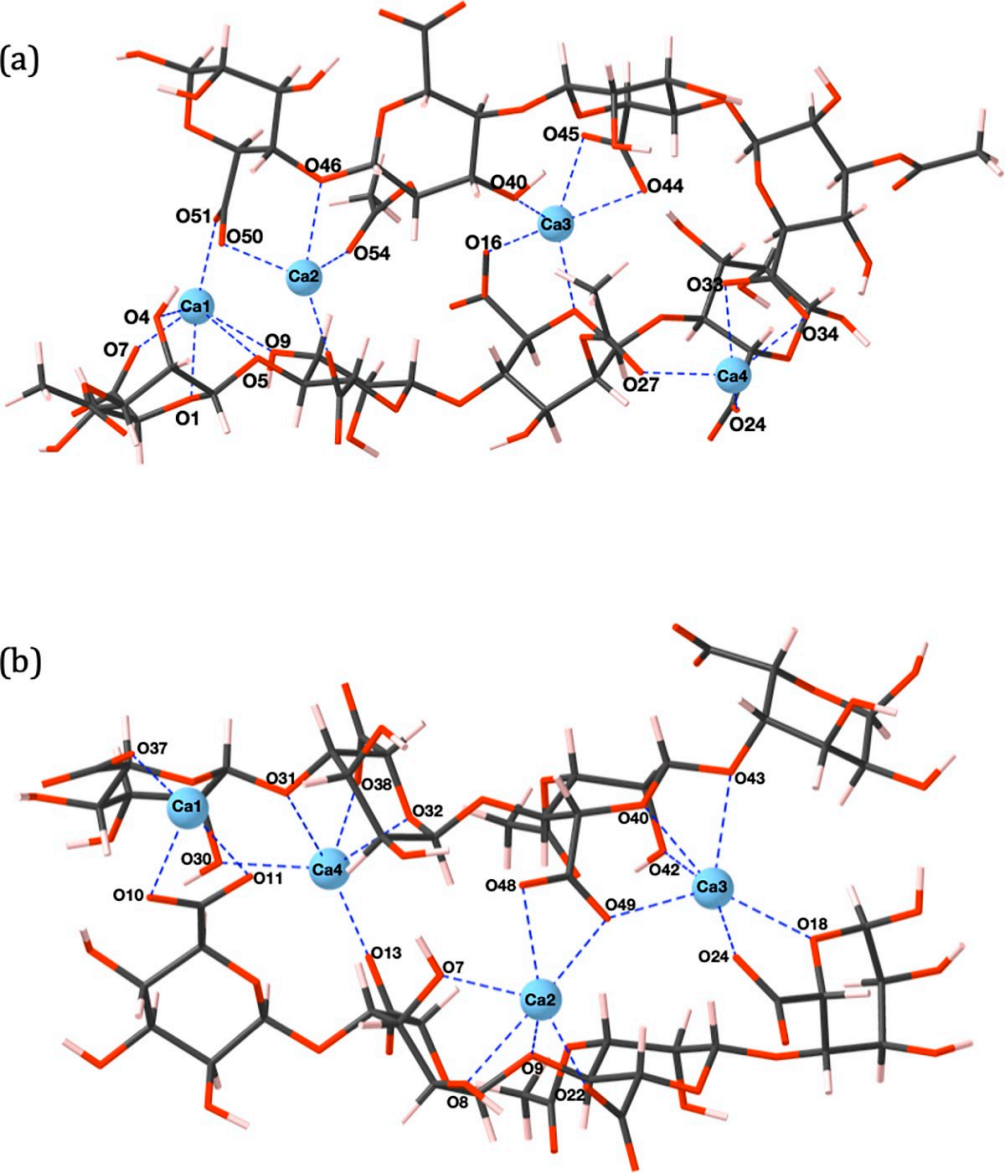
(a) PolyM_(ap)_ (b) PolyMG_(p)_ 2-chain calcium complexes. Carbon atoms are shown in grey, oxygen in red, calcium in blue and hydrogen in pink. Oxygen atoms and cations involved in ionic bonding are labelled and the cation-oxygen bonds are shown with dashed blue lines.

**Fig 4:**
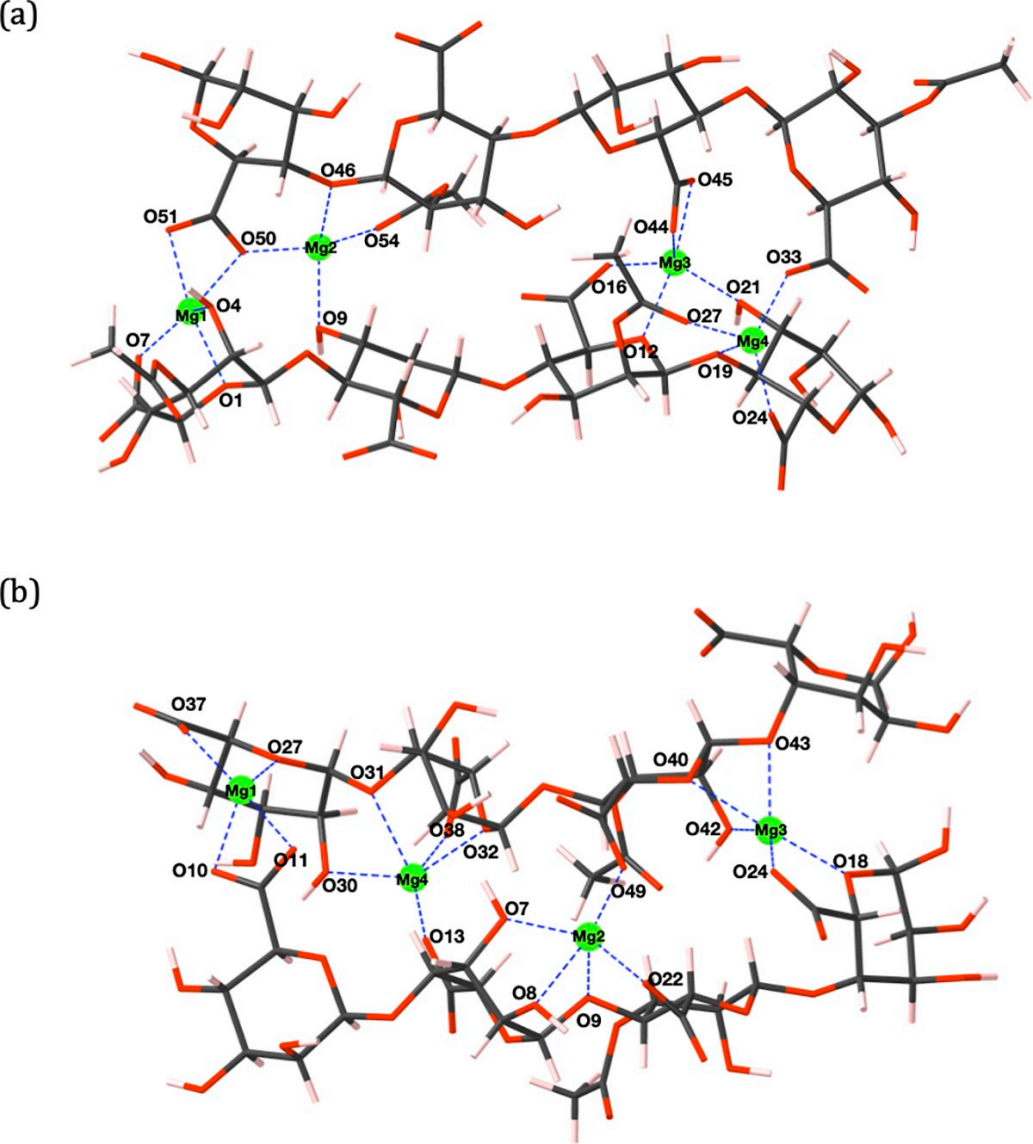
(a) PolyM_(ap)_ (b) PolyMG_(p)_ 2-chain magnesium complexes. Carbon atoms are shown in grey, oxygen in red, magnesium in green and hydrogen in pink. Oxygen atoms and cations involved in ionic bonding are labelled and the cation-oxygen bonds are shown with dashed blue lines.

All cations preferentially bind in positions whereby multiple oxygen contacts are sustained. Calcium (Fig 3) and magnesium (Fig 4) ions adopt coordination environments displaying analogies to the *egg-box model* of divalent ion complexation by algal alginates [55], namely, the formation of chelate pockets formed in-between adjacent chains. However, the chelate complex geometries adopted by Ca^2+^ and Mg^2+^ ions do not entirely match the geometries predicted by the egg-box model. Specifically, egg-box binding of divalent cations is facilitated through the establishment of ten ionic contacts, five from each polyuronate chain, where four uronate residues are responsible for binding a single divalent cation [55]. The coordination geometries highlighted in this work have 2–3 uronate residues per divalent ion and a maximum coordination number < 10. In fact, in the work of Braccini *et al*., moving a Ca^2+^ probe over the Van der Waals (VdW) surface of poly-*β*-D-mannuronate and poly-*α*-L-guluronate hexamers showed a wide variety of possible binding positions for Ca^2+^ ions within a 15 kcal/mol energetic window, where the only prerequisite was the suitable orientation of oxygen atoms [49]. Deviations away from the egg-box model have also been observed in MD studies where 2-chain associations have been created solely through Ca^2+^-COO^-^ interactions [32,33], and through perpendicular chain conformations [35].

**Fig 5:**
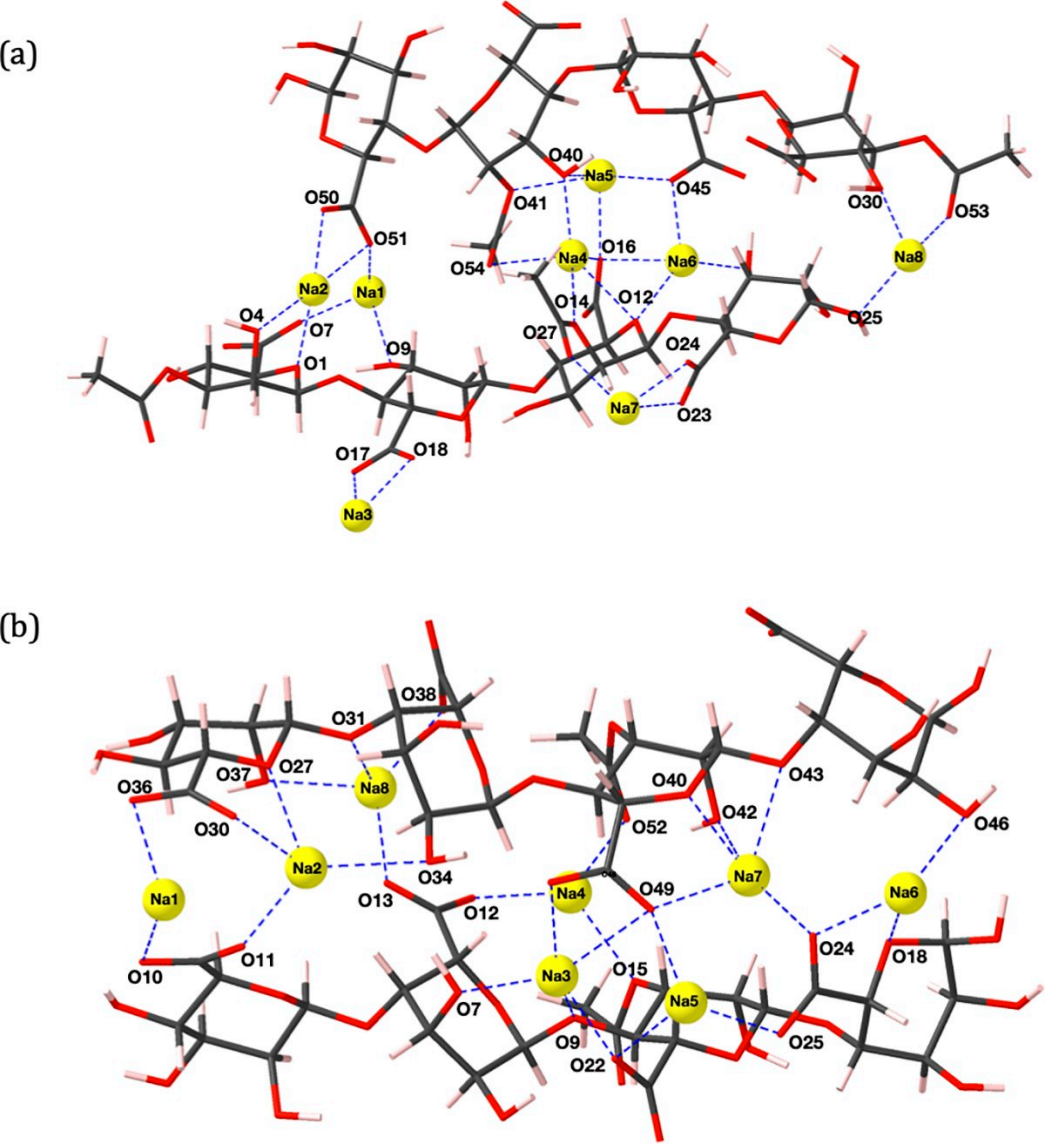
(a) PolyM_(ap)_ (b) PolyMG_(p)_ 2-chain sodium complexes. Carbon atoms are shown in grey, oxygen in red, sodium in yellow and hydrogen in pink. Oxygen atoms and cations involved in ionic bonding are labelled and the cation-oxygen bonds are shown with dashed blue lines.

For both the calcium and magnesium cross-linked polyuronate systems, two COO^-^ groups are responsible for binding a single cation, in agreement with predictions from thermogravimetric experiments investigating divalent cation complexation by algal alginate [56]. In contrast, sodium ions do not present defined binding regions as, due to the stoichiometry of the system, single COO^-^ groups are involved in binding multiple sodium ions (Fig 5). Cooperative behaviour, the principle that the polyuronate chain offers defined binding sites distributed in regular arrays, is prevalent in divalent but absent in monovalent ion complexation. Ill-defined inter-chain sodium ion binding positions led to thermodynamically unfavourable complexation, *E*_*f*_ = +0.62 eV (Table 2), in the PolyM_(ap)_ system (Fig 5A). This unfavourability can be attributed to two sodium ions (Na3 and Na7) binding to outward facing COO^-^ groups (O17, O18, O23 and O24) that do not contribute to binding of the two chains. In addition, Na8 binds in a position that fails to bind to a COO^-^ group because it binds to terminal acetyl and hydroxyl groups (O25, O30 and O53) at the periphery of the terminal residue. Similar binding modes have been shown in previous MD simulations of 10-unit poly-*β*-D-mannuronate chains. In these simulations the sodium failed to show any preferential binding for the carboxylate group and formed only transient interactions with other oxygen atoms, such that inter-chain cation-mediated cross-linking was not observed [36].

Coordination numbers of up to six are present in the calcium 2-chain complexes, which matches the number of contacts in optimized structures observed in a previous DFT study on calcium complexation by two algal alginate hexamers [38]. In both polyuronate systems, the calcium and magnesium ions bind in-between the chains and mediate cross-links; binding externally in either system is not observed. However, the maximum coordination number (CN) around the magnesium cation (CN = 5) is lower than for calcium (CN = 6), despite the higher charge density of magnesium. Similar observations have been previously reported for M-G diuronate cation complexes [50] and can be explained by the smaller ionic radius of Mg^2+^, which reduces the number of accessible oxygen atoms [57]. Considering the PolyM_(ap)_ system, Ca1 (Fig 3A) and Mg1 (Fig 4A) adopt similar coordination environments, apart from Ca1-O9, which is absent in the magnesium complex. Moreover, in the PolyMG_(p)_ system, Mg2 and Mg3 (Fig 4B) coordination environments are similar to Ca2 and Ca3 (Fig 3B) other than the Mg2-O48 and Mg3-O49 interactions being absent. Consequently, magnesium complexes are less thermodynamically stable compared to the calcium complexes, which helps to explain the poor gelation ability of magnesium ions [58]. It is worth noting that in a previous DFT study on a single algal alginate M-G junction, magnesium was shown to complex with *greater* stability relative to calcium [39]. This highlights the importance of employing a model constructed from multiple M-M/M-G junctions (from opposing chains) to predict the effect that ionic radii have on uronate oxygen accessibility and to capture the correct gelation trends.

### Analysis of bonding architecture

By considering CN, electronic spin state and cation-anion interatomic separation, Shannon and Prewitt-derived ionic radii are appropriate for systems where bonding of cations occurs to oxygen [59]. For the sputum ions considered in this work, the trend in ionic radii is Na^+^ ~ Ca^2+^ > Mg^2+^ across an array of different coordination environments. Cation-oxygen bond lengths (see S3–S5 Tables) follow a trend that reflects the trend in ionic radii. Specifically, the average Na^+^-oxygen length (2.36 Å) ~ the average Ca^2+^-oxygen length (2.35 Å) > the average Mg^2+^-oxygen length (2.03 Å). The Ca^2+^-oxygen and Mg^2+^-oxygen bond lengths agree well (within 4–8%) with observations from previous DFT calculations on divalent cation complexation by M-M and G-G diuronates [37].

Mulliken bond populations (see S3–S5 Tables) show that all cation-oxygen bonds, across all 2-chain complexes, are ionic, defined by cation-oxygen populations < 0.3 |e|. The average cation-COO^-^ bond lengths for Na^+^, Ca^2+^ and Mg^2+^ in the PolyM_(ap)_ system are 2.32 Å, 2.26 Å and 1.99 Å respectively and in the PolyMG_(p)_ system they are 2.27 Å, 2.26 Å and 1.94 Å respectively. These are the shortest of all the cation-oxygen contacts and indicate that there is a stronger interaction between the cations and the COO^-^ groups compared with other oxygen functionality. The average Na^+^-COO^-^ populations (PolyM_(ap)_ 0.06 |e|, PolyMG_(p)_ 0.08 |e|) are smaller compared with the average Ca^2+^-COO^-^ (PolyM_(ap)_ 0.14 |e|, PolyMG_(p)_ 0.14 |e|) and Mg^2+^-COO^-^ (PolyM_(ap)_ 0.13 |e|, PolyMG_(p)_ 0.17 |e|) populations, indicating the strength of the cation-COO^-^ interaction is considerably larger for calcium and magnesium ions, which is to be expected following charge density arguments.

Furthermore, comparing the PolyM_(ap)_ and PolyMG_(p)_ 2-chain complexes for each sputum ion, fewer COO^-^ groups are saturated in the sodium and magnesium PolyM_(ap)_ system, compared to their PolyMG_(p)_ analogues. In these instances, the formation energy (Table 3) difference between the two systems is ~2.2 eV for sodium and ~ 1.7 eV for magnesium, with PolyMG_(P)_ always having the lower formation energy. In the case of calcium, the same number of COO^-^ groups are saturated in both polyuronate systems and, subsequently, the formation energy difference is lower (~ 0.5 eV). Therefore, within the slight preference for binding M-G-blocks, there is also a stabilizing effect of COO^-^ saturation.

All these factors highlight the electrostatic nature of cation complexation by the mucoid *P*. *aeruginosa* EPS. Comparing the calcium and magnesium 2-chain complexes, the atomic charges on Ca and Mg cations range from 1.44–1.55 and 1.55–1.66 respectively. Moreover, the magnitude of the average Mg^2+^-COO^-^ populations (PolyM_(ap)_ 0.13 |e|, PolyMG_(p)_ 0.17 |e|), although near equivalent in the PolyM_(ap)_ complexes, exceed the Ca^2+^-COO^-^ populations in the PolyMG_(p)_ complexes by 0.03 |e|. The atomic charge and bond populations imply that, using electrostatic arguments, magnesium ions should give more thermodynamically stable 2-chain complexes. However, this is not observed. It is clear that, although electrostatics play a role in cation complexation, they do not dictate complex stability.

The cross-linked complexes accommodate the number of cations required to saturate the total negative charge of the carboxylate groups, assuming mono and divalent ion charges for sodium and magnesium/calcium respectively. Under these conditions, sodium ions display ill-defined inter-chain binding sites and single COO^-^ groups are involved in ionic bonding to multiple sodium ions (Fig 5). The consequence of this is a bonding architecture constructed from many weak ionic bonds, shown by Na^+^-COO^-^ bond populations between 0.03–0.09 |e| (see S3 Table), and external binding giving fewer cross-linking interactions (Fig 5A). This effect is not prevalent in the calcium and magnesium 2-chain complexes, which display more defined binding sites. Differences in stability between these complexes can be attributed to lower coordination numbers adopted by magnesium ions due to their smaller ionic radius. Furthermore, larger global changes in the dihedral angles (ϕ, ψ) are observed in the divalent ion 2-chain complexes compared to the monovalent ion 2-chain complexes (S1 Table). Upon complexing Na^+^ ions, deviations in ϕ or ψ do not surpass 39˚ in both PolyM_(ap)_ and PolyMG_(p)_ 2-chain systems. However, upon complexing Ca^2+^ and Mg^2+^ ions, deviations reach 49˚ in the PolyMG_(p)_ 2-chain systems and 313˚ in the PolyM_(ap)_ 2-chain systems. The large changes in the dihedral angles in the PolyM_(ap)_ divalent ion complexes are required to reorient the glycosidic linkage at O46 to face Ca2 and Mg2, establishing an ionic contact (Figs 3A and 4A). This is not observed with PolyM_(ap)_ complexing Na^+^ ions. As such, calcium and magnesium ions have greater influence on the polyuronate conformation compared to sodium ions, which has also seen in the complexes established between Na^+^, Ca^2+^ and Mg^2+^ ions with M-M, M-G, G-M and G-G diuronates [39]. Moreover, larger torsional changes in the divalent ion PolyM_(ap)_ complexes indicate that M-G junctions offer oxygen functionality whose spatial arrangement is suitable for saturating the coordination environment of the cation. Similar observations have been made in molecular mechanics studies on the conformational features of acidic polysaccharides interacting with calcium ions [49]. Therefore, it is likely that the requirement for large torsional change in the PolyM_(ap)_ divalent ion complexes is another reason for the preference for binding within M-G blocks and the greater thermodynamic stability of the PolyMG_(p)_ divalent ion complexes. This is further evidence of cooperative behaviour—the chain must adopt a conformation that creates stable defined binding positions for the (divalent) cations. Overall, the stability of the 2-chain complexes are, more significantly, determined by the geometry of the chelation site rather than the strength of the cation-oxygen bonds. This, in turn, is dependent on steric factors, namely, defined inter-chain binding sites and uronate oxygen accessibility. We clearly show that such steric effects can only be captured by employing a molecular model of a large enough molecular weight, encompassing multiple M-M/M-G junctions from opposing chains.

Although sodium ions are ubiquitous in human physiology, and are consequently the most abundant metal ion in CF sputum, it is clear that sodium is not implicated in the aggregation of the mucoid *P*. *aeruginosa* extracellular polysaccharide or the ECM stability. By comparison, both calcium and magnesium ions, which are greatly elevated in CF sputum, have been shown to have a direct effect on extracellular polysaccharide aggregation and consequently, ECM stability. Calcium ions are predicted to induce the most stable aggregation of the EPS and can therefore be considered the most important sputum ion, out of those tested, for mucoid *P*. *aeruginosa* ECM stability and biofilm chronicity.

## Conclusions

In this work, the relationship between structural chemistry and bacterial virulence has been probed in detail for mucoid *P*. *aeruginosa* extracellular polysaccharide molecular systems. Specifically, two two-chain mucoid *P*. *aeruginosa* extracellular polysaccharide molecular models were constructed, representing areas of zero and 50% guluronate as well possessing acetyl groups–an *in vivo* structural motif unique to mucoid *P*. *aeruginosa*. Thus, these models are, uniquely, structurally representative of the mucoid biofilm exopolysaccharide architecture. Precise step-wise building of the models ensured we had the most accurate system constructed to date.

We demonstrated that stable accommodation of sputum ions by the mucoid *P*. aeruginosa EPS is electrostatic in origin but the stability of the resulting complex is, in fact, influenced more by the geometry of the chelation sites rather than simply the strength of the cation-oxygen bonds. In regions where guluronate units are absent and in regions where they are in their highest possible abundance (represented by the PolyM_(ap)_ and PolyMG_(p)_ systems respectively), calcium ions are able to produce cross-linked structures ~9 eV and ~5 eV more thermodynamically stable relative to sodium and magnesium ions. These are large differences, which show unequivocally how the chemistry of these two ions differs from that of the biologically ubiquitous sodium ion, and impacts upon the stability of the EPS structure. These observations are important in providing new chemical insight into previous experimental reports of thicker biofilms being produced in the presence of excess calcium [27–29].

In regions of guluronate inclusion, more thermodynamically stable cross-linked structures were obtained, highlighting a preference for binding within M-G blocks where preferential binding sites can be achieved without the large torsional rotations required by the PolyM system. This demonstrates well the importance of using larger whole-chain models rather than relying on smaller subunits to infer chemical nuance, and clearly explains the significance of the guluronate units in the alginate chains, with regard to bacterial virulence.

The greater stability of the calcium 2-chain complexes rationalizes the virulent consequences of mucoid *P*. *aeruginosa* ECM exposure to calcium ions, namely the development of thicker, more granular and rigid biofilms that are difficult to detach [27–29]. Possible strategies to combat mucoid biofilm infection in the cystic fibrosis lung may therefore include calcium chelation. Indeed, recently, Powell *et al*. have demonstrated that a low molecular weight guluronate rich alginate oligomer, which binds calcium ions, can disrupt established biofilms [60]. From the work presented here, it is clear that preventing calcium ions from mediating electrostatic cross-links between bacterial alginate structures will be critical in facilitating the disruption of the mucoid biofilms and that using sufficiently detailed theoretical models is necessary for establishing meaningful chemical insight. Indeed, these cation cross-linked exopolysaccharide structures will, in the future, serve as precise model systems to study other cation substitutions and small-molecule pharmaceutical interventions at the molecular scale.

## Supporting information

S1 Fig Most thermodynamically stable binding position for single Na+ ion along the length of a single PolyM (top) and PolyMG (bottom) chain. Carbon atoms are shown in grey, oxygen in red, sodium in yellow and hydrogen in pink. Ionic bonds to the sodium ion are labelled as blue dash lines. For reference, the optimized positions of the ions at the other two points along the length of the chain axis are labelled and displayed as grey balls.
(TIF)

S2 Fig Most thermodynamically stable binding position for single Ca2+ ion along the length of a single PolyM (top) and PolyMG (bottom) chain. Carbon atoms are shown in grey, oxygen in red, calcium in blue and hydrogen in pink. Ionic bonds to the calcium ion are labelled as blue dash lines. For reference, the optimized positions of the ions at the other two points along the length of the chain axis are labelled and displayed as grey balls.
(TIF)

S3 Fig Most thermodynamically stable binding position for single Mg2+ ion along the length of a single PolyM (top) and PolyMG (bottom) chain. Carbon atoms are shown in grey, oxygen in red, magnesium in green and hydrogen in pink. Ionic bonds to the magnesium ion are labelled as blue dash lines. For reference, the optimized positions of the ions at the other two points along the length of the chain axis are labelled and displayed as grey balls.
(TIF)

S1 Table Dihedral angles (ϕ,ψ) for the polyuronate systems. All angles are given in degrees (^O^). See Fig 1 for labelling of the uronate units.
(DOCX)

S2 Table Formation energies (eV) for all the single ion binding positions along the length of a single PolyM and PolyMG chain. Formation energies for the fully charge-saturated single PolyM and PolyMG chains with respect to the four carboxylic acid groups are also given.
(DOCX)

S3 Table Bond populations and lengths for the Na^+^-oxygen contacts in the sodium 2-chain complexes. (DOCX)

S4 Table Bond populations and lengths for the Ca^2+^-oxygen contacts in the calcium 2-chain complexes. (DOCX)

S5 Table Bond populations and lengths for the Mg^2+^-oxygen contacts in the magnesium 2-chain complexes. (DOCX)
