# Supplementary figures and images for: Cation complexation by mucoid Pseudomonas aeruginosa extracellular polysaccharide
Source: PLoS One. 2021 Sep 2;16(9):e0257026. doi: 10.1371/journal.pone.0257026 (PMC8412252; doi:10.1371/journal.pone.0257026)

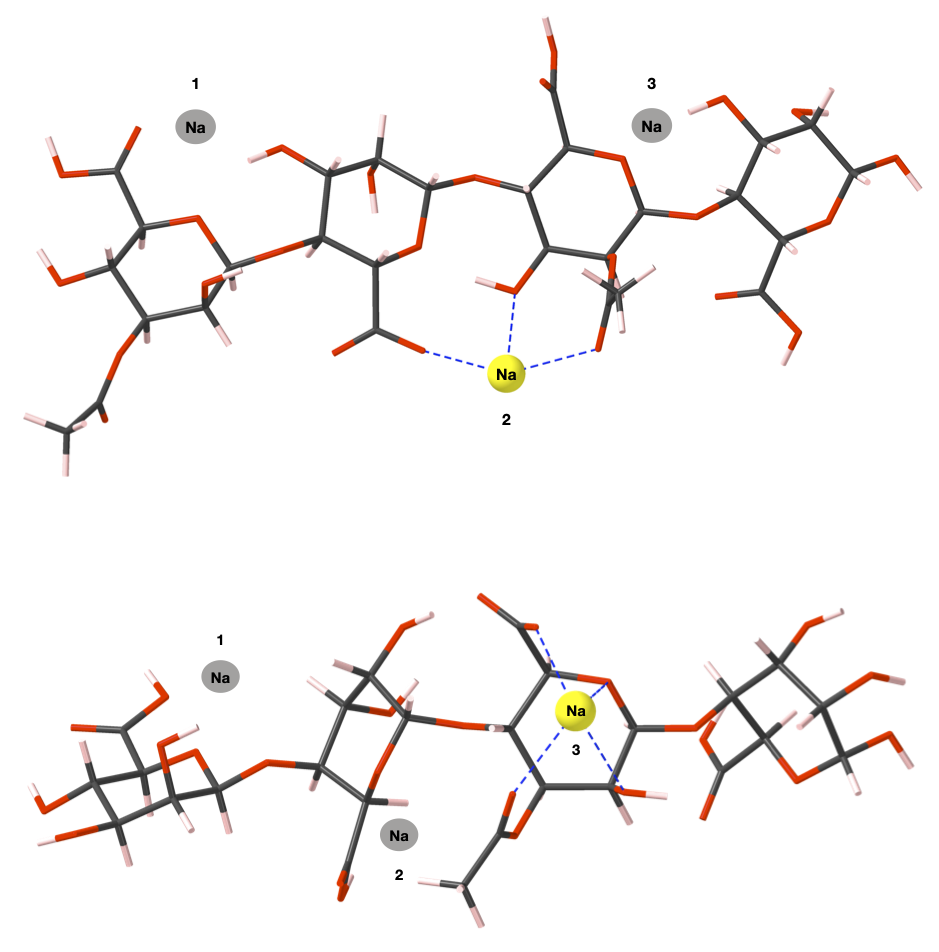

Supplement: S1 Fig — Most thermodynamically stable binding position for single Na+ ion along the length of a single PolyM (top) and PolyMG (bottom) chain. Carbon atoms are shown in grey, oxygen in red, sodium in yellow and hydrogen in pink. Ionic bonds to the sodium ion are labelled as blue dash lines. For reference, the optimized positions of the ions at the other two points along the length of the chain axis are labelled and displayed as grey balls. (TIF) [file pone.0257026.s001.tif]

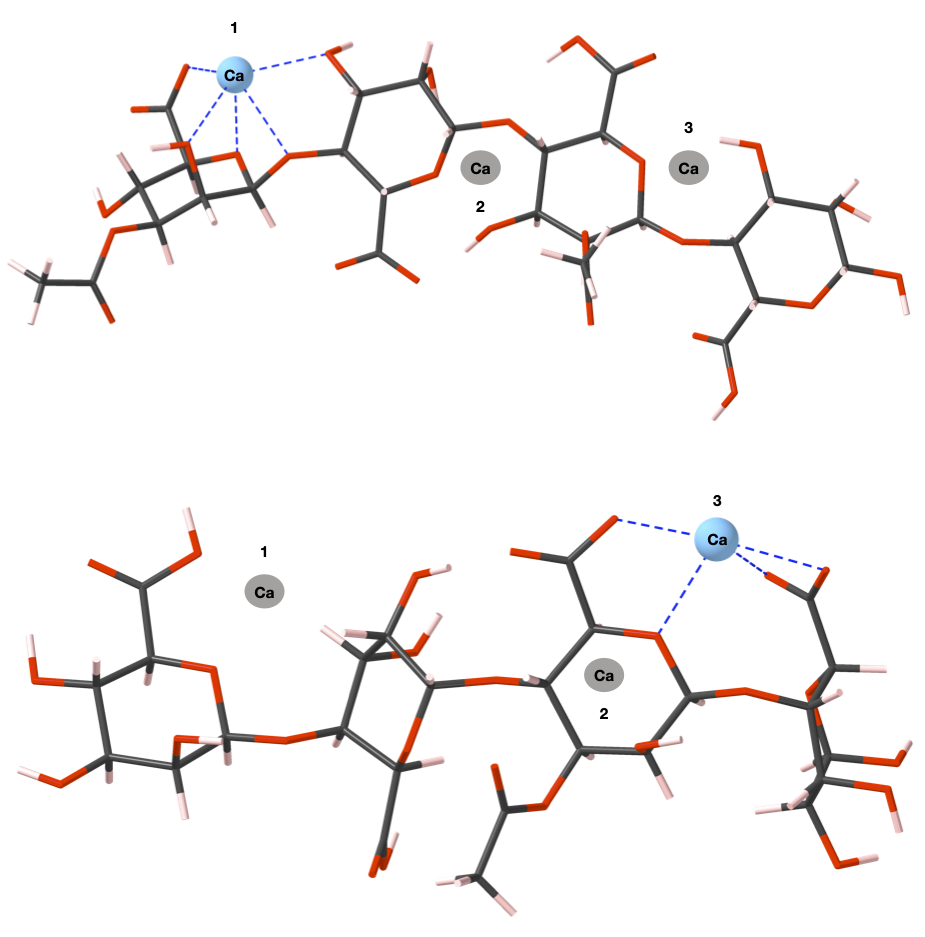

Supplement: S2 Fig — Most thermodynamically stable binding position for single Ca2+ ion along the length of a single PolyM (top) and PolyMG (bottom) chain. Carbon atoms are shown in grey, oxygen in red, calcium in blue and hydrogen in pink. Ionic bonds to the calcium ion are labelled as blue dash lines. For reference, the optimized positions of the ions at the other two points along the length of the chain axis are labelled and displayed as grey balls. (TIF) [file pone.0257026.s002.tif]

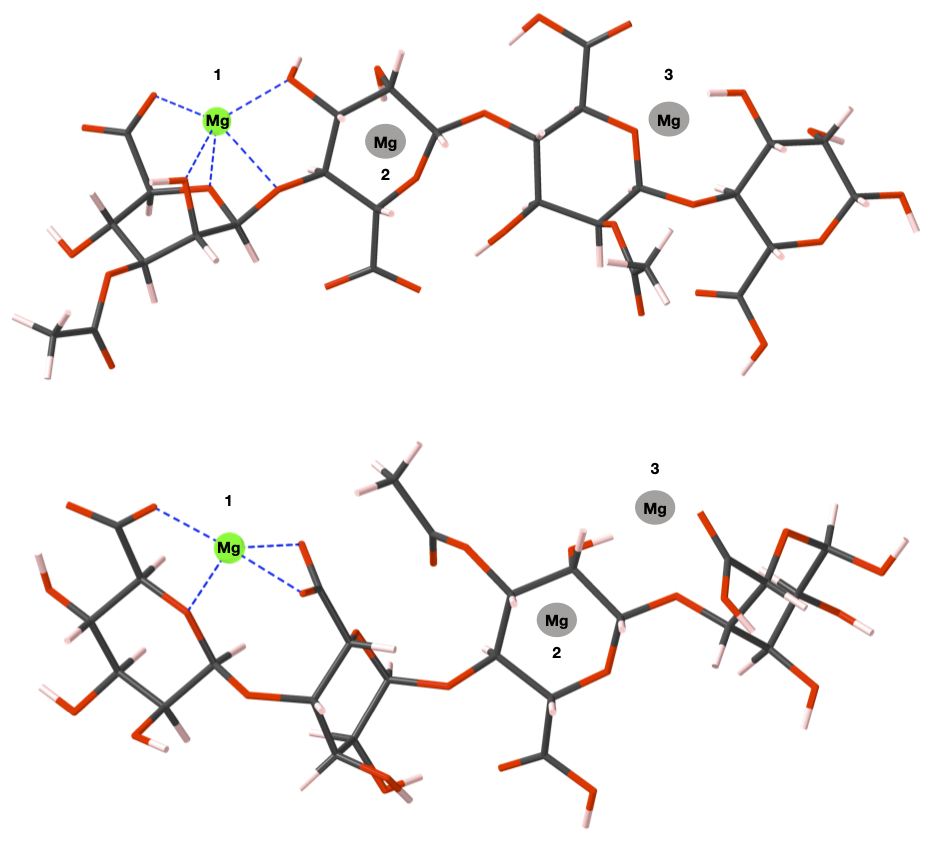

Supplement: S3 Fig — Most thermodynamically stable binding position for single Mg2+ ion along the length of a single PolyM (top) and PolyMG (bottom) chain. Carbon atoms are shown in grey, oxygen in red, magnesium in green and hydrogen in pink. Ionic bonds to the magnesium ion are labelled as blue dash lines. For reference, the optimized positions of the ions at the other two points along the length of the chain axis are labelled and displayed as grey balls. (TIF) [file pone.0257026.s003.tif]
