# Supplementary material for: Cation complexation by mucoid Pseudomonas aeruginosa extracellular polysaccharide
Source: PLoS One. 2021 Sep 2;16(9):e0257026. doi: 10.1371/journal.pone.0257026 (PMC8412252; doi:10.1371/journal.pone.0257026)
Supplement: S1 Table — All angles are given in degrees (O). See Fig 1 for labelling of the uronate units. (DOCX) [file pone.0257026.s004.docx]

**Cation Complexation by Mucoid *Pseudomonas aeruginosa* Extracellular Polysaccharide**

Oliver J. Hills, James Smith, Andrew Scott, Deirdre A. Devine & Helen F. Chappell

**Supplementary information**

**Dihedral angles for all polyuronate systems**

Dihedral angles $(\phi, \psi)$ for the polyuronate molecular models and polyuronate-ion complexes are given. For systems possessing two polyuronate chains, dihedral angles for the glycosidic bonds in the top and bottom chains are given.

**Table 1**: Dihedral angles $(\phi,\psi)$ for the polyuronate systems. All angles are given in degrees (^0^). See Figure 1 for labelling of the uronate units.

| System | M1M2/M1G1 | M2M3/G1M2 | M3M4/M2G2 |
| --- | --- | --- | --- |
| PolyM | (-76, -132) | (-97, -155) | (-85, -134) |
| PolyMG | (-68, -114) | (-95, -146) | (-74, -100) |
| PolyM_(ap)_ | Top chain: (-70, -125)  Bottom chain: (-54, -130) | Top chain: (-70, -134)  Bottom chain: (-94, -156) | Top chain: (-96, -151)  Bottom chain: (-107, -123) |
| PolyMG_(p)_ | Top chain: (-87, -139)  Bottom chain: (-67, -132) | Top chain: (-91, -137)  Bottom chain: (-101, -144) | Top chain: (-74, -99)  Bottom chain: (-47, -55) |
| Na-PolyM_(ap)_ | Top chain: (-61, -120)  Bottom chain: (-81, -139) | Top chain: (-43, -121)  Bottom chain: (-95, -136) | Top chain: (-78, -171)  Bottom chain: (-111, -118) |
| Ca-PolyM_(ap)_ | Top chain: (-51, -99)  Bottom chain: (-76, -121) | Top chain: (-51, -127)  Bottom chain: (-93, -103) | Top chain: (-49, +139)  Bottom chain: (-102, -108) |
| Mg-PolyM_(ap)_ | Top chain: (-49, -67)  Bottom chain: (-81, -102) | Top chain: (-77, -125)  Bottom chain: (-103, -142) | Top chain: (-72, +162)  Bottom chain: (-85, -123) |
| Na-PolyMG_(p)_ | Top chain: (-85, -155)  Bottom chain: (-49, -116) | Top chain: (-88, -140)  Bottom chain: (-107, -143) | Top chain: (-113, -79)  Bottom chain: (-48, -65) |
| Ca-PolyMG_(p)_ | Top chain: (-91, -127)  Bottom chain: (-58, -122) | Top chain: (-94, -130)  Bottom chain: (-89, -130) | Top chain: (-110, -78)  Bottom chain: (-64, -44) |
| Mg-PolyMG_(p)_ | Top chain: (-104, -106)  Bottom chain: (-50, -84) | Top chain: (-93, -128)  Bottom chain: (-92, -137) | Top chain: (-123, -61)  Bottom chain: (-55, -48) |
