# Supplementary material for: Cation complexation by mucoid Pseudomonas aeruginosa extracellular polysaccharide
Source: PLoS One. 2021 Sep 2;16(9):e0257026. doi: 10.1371/journal.pone.0257026 (PMC8412252; doi:10.1371/journal.pone.0257026)
Supplement: S2 Table — Formation energies for the fully charge-saturated single PolyM and PolyMG chains with respect to the four carboxylic acid groups are also given. (DOCX) [file pone.0257026.s005.docx]

**Cation Complexation by Mucoid *Pseudomonas aeruginosa* Extracellular Polysaccharide**

Oliver J. Hills, James Smith, Andrew Scott, Deirdre A. Devine & Helen F. Chappell

**Supplementary information**

**Single ion complexes**

The most thermodynamically favourable binding position of a single ion along the length of a single PolyM and PolyMG chain. Table 2 displays the formation energy for each single ion complex and charge saturated complexes. Charge saturating a single chain with respect to all carboxylic acid groups gave more thermodynamically stable structures relative to the single ion complexes.

**Table 2**: Formation energies (eV) for all the single ion binding positions along the length of a single PolyM and PolyMG chain. Formation energies for the fully charge-saturated single PolyM and PolyMG chains with respect to the four carboxylic acid groups are also given.

| PolyM | | |
| --- | --- | --- |
| Cation | Position | Formation Energy ($E_{f}$), eV |
| Na | 1 | -0.97 |
|  | 2 | -1.15 |
|  | 3 | -0.13 |
|  | Charge saturated | -3.55 |
| Ca | 1 | -1.81 |
|  | 2 | -1.17 |
|  | 3 | -1.26 |
|  | Charge saturated | -3.04 |
| Mg | 1 | -0.49 |
|  | 2 | +0.24 |
|  | 3 | +0.08 |
|  | Charge saturated | -0.65 |
| PolyMG | | |
| Na | 1 | -0.89 |
|  | 2 | -1.21 |
|  | 3 | -1.35 |
|  | Charge saturated | -4.58 |
| Ca | 1 | -0.94 |
|  | 2 | -0.84 |
|  | 3 | -2.94 |
|  | Charge saturated | -5.73 |
| Mg | 1 | -1.85 |
|  | 2 | -0.10 |
|  | 3 | -1.84 |
|  | Charge saturated | -3.53 |
