# Supplementary material for: Cation complexation by mucoid Pseudomonas aeruginosa extracellular polysaccharide
Source: PLoS One. 2021 Sep 2;16(9):e0257026. doi: 10.1371/journal.pone.0257026 (PMC8412252; doi:10.1371/journal.pone.0257026)
Supplement: S3 Table — (DOCX) [file pone.0257026.s006.docx]

**Cation Complexation by Mucoid *Pseudomonas aeruginosa* Extracellular Polysaccharide**

Oliver J. Hills, James Smith, Andrew Scott, Deirdre A. Devine & Helen F. Chappell

**Supplementary information**

Mulliken bond populations and bond lengths for all Na^+^-oxygen contacts in each polyuronate-ion complex is given in Tables 3. For each contact, the oxygen functional group is indicated.

**Table 3**: Bond populations and lengths for the Na^+^-oxygen contacts in the sodium 2-chain complexes.

| Sodium PolyM_(ap)_ complex | | |
| --- | --- | --- |
| Bond | Population (\|e\|) | Length (Å) |
| Na1-O7 (COO^-^)  Na1-O9 (OH)  Na1-O51 (COO^-^)  Na2-O1 (Ring O)  Na2-O4 (OH)  Na2-O50 (COO^-^)  Na2-O51(COO^-^)  Na3-O17 (COO^-^)  Na3-O18 (COO^-^)  Na4-O12 (Ring O)  Na4-O14 (Acetyl)  Na4-O16 (COO^-^)  Na4-O40 (OH)  Na4-O54 (Acetyl)  Na5-O16 (COO^-^)  Na5-O40 (OH)  Na5-O41 (Acetyl)  Na5-O45 (COO^-^)  Na6-O12 (Ring O)  Na6-O16 (COO^-^)  Na6-O21 (OH)  Na6-O45 (COO^-^)  Na7-O23 (COO^-^)  Na7-O24 (COO^-^)  Na7-O27 (Acetyl)  Na8-O25 (OH)  Na8-O30 (OH)  Na8-O53 (Acetyl) | 0.13  0.1  0.05  0.07  0.05  0.02  0.03  0.04  0.05  0.07  0.11  0.09  0.12  0.06  0.07  0.01  0.09  0.11  0.06  0.03  0.04  0.07  0.05  0.04  0.06  0.05  0.14  0.06 | 2.15  2.19  2.38  2.46  2.36  2.25  2.46  2.24  2.24  2.63  2.5  2.43  2.26  2.63  2.34  2.97  2.53  2.27  2.31  2.37  2.3  2.256  2.34  2.23  2.29  2.33  2.16  2.28 |
| Sodium PolyMG_(p)_ complex | | |
| Bond | Population (\|e\|) | Length (Å) |
| Na1-O10 (COO^-^)  Na1-O36 (COO^-^)  Na2-O11 (COO^-^)  Na2-O27 (Ring O)  Na2-O34 (OH)  Na2-O37 (COO^-^)  Na3-O7 (OH)  Na3-O9 (Glycosidic O)  Na3-O22 (COO^-^)  Na3-O48 (COO^-^)  Na4-O12 (COO^-^)  Na4-O15 (Acetyl)  Na4-O52 (Acetyl)  Na5-O22 (COO^-^)  Na5-O25 (COO^-^)  Na5-O49 (COO^-^)  Na6-O18 (Ring O)  Na6-O24 (COO^-^)  Na6-O46 (OH)  Na7-O24 (COO^-^)  Na7-O40 (Ring O)  Na7-O42 (OH)  Na7-O43 (Glycosidic O)  Na7-O49 (COO^-^)  Na8-O13 (COO^-^)  Na8-O30 (OH)  Na8-O31 (Glycosidic O)  Na8-O38 (COO^-^) | 0.03  0.02  0.12  0.11  0.08  0.11  0.06  0.12  0.11  0.05  0.13  0.09  0.08  0.07  0.09  0.06  0.08  0.08  0.06  0.08  0.05  0.11  0.1  0.1  0.06  0.07  0.08  0.1 | 2.34  2.37  2.21  2.63  2.68  2.25  2.22  2.53  2.25  2.3  2.16  2.38  2.28  2.29  2.3  2.32  2.44  2.21  2.37  2.22  2.29  2.43  2.52  2.39  2.22  2.43  2.49  2.24 |
