# Supplementary material for: Cation complexation by mucoid Pseudomonas aeruginosa extracellular polysaccharide
Source: PLoS One. 2021 Sep 2;16(9):e0257026. doi: 10.1371/journal.pone.0257026 (PMC8412252; doi:10.1371/journal.pone.0257026)
Supplement: S4 Table — (DOCX) [file pone.0257026.s007.docx]

**Cation Complexation by Mucoid *Pseudomonas aeruginosa* Extracellular Polysaccharide**

Oliver J. Hills, James Smith, Andrew Scott, Deirdre A. Devine & Helen F. Chappell

**Supplementary information**

Mulliken bond populations and bond lengths for all Ca^2+^-oxygen contacts in each polyuronate-ion complex is given in Table 4. For each contact, the oxygen functional group is indicated.

**Table 4**: Bond populations and lengths for the Ca^2+^-oxygen contacts in the calcium 2-chain complexes.

| Calcium PolyM_(ap)_ complex | | |
| --- | --- | --- |
| Bond | Population (\|e\|) | Length (Å) |
| Ca1-O1 (Ring O)  Ca1-O4 (OH)  Ca1-O5 (Glycosidic O)  Ca1-O7 (COO^-^)  Ca1-O9 (OH)  Ca1-O51 (COO^-^)  Ca2-O18 (COO^-^)  Ca2-O46 (Glycosidic O)  Ca2-O50 (COO^-^)  Ca2-O54 (Acetyl)  Ca3-O12 (Ring O)  Ca3-O16 (COO^-^)  Ca3-O40 (OH)  Ca3-O44 (COO^-^)  Ca3-O45 (COO^-^)  Ca4-O24 (COO^-^)  Ca4-O27 (Acetyl)  Ca4-O33 (COO^-^)  Ca4-O34 (COO^-^) | 0.08  0.09  0.04  0.19  0.09  0.11  0.21  0.12  0.13  0.11  0.11  0.18  0.09  0.06  0.09  0.22  0.1  0.08  0.08 | 2.39  2.48  2.68  2.17  2.46  2.42  2.15  2.46  2.24  2.33  2.41  2.18  2.46  2.42  2.34  2.1  2.31  2.32  2.34 |
| Calcium PolyMG_(p)_ complex | | |
| Bond | Population (\|e\|) | Length (Å) |
| Ca1-O10 (COO^-^)  Ca1-O11 (COO^-^)  Ca1-O37 (COO^-^)  Ca2-O7 (OH)  Ca2-O8 (OH)  Ca2-O9 (Glycosidic O)  Ca2-O22 (COO^-^)  Ca2-O48 (COO^-^)  Ca2-O49 (COO^-^)  Ca3-O18 (Ring O)  Ca3-O24 (COO^-^)  Ca3-O40 (Ring O)  Ca3-O42 (OH)  Ca3-O43 (Glycosidic O)  Ca3-O49 (COO^-^)  Ca4-O13 (COO^-^)  Ca4-O30 (OH)  Ca4-O31 (Glycosidic O)  Ca4-O32 (Ring O)  Ca4-O38 (COO^-^) | 0.1  0.09  0.2  0.1  0.06  0.1  0.19  0.05  0.06  0.12  0.21  0.08  0.1  0.08  0.09  0.24  0.1  0.08  0.09  0.16 | 2.23  2.25  2.08  2.37  2.46  2.46  2.20  2.52  2.42  2.52  2.13  2.41  2.41  2.59  2.53  2.11  2.41  2.56  2.44  2.20 |
