# Supplementary material for: Cation complexation by mucoid Pseudomonas aeruginosa extracellular polysaccharide
Source: PLoS One. 2021 Sep 2;16(9):e0257026. doi: 10.1371/journal.pone.0257026 (PMC8412252; doi:10.1371/journal.pone.0257026)
Supplement: S5 Table — (DOCX) [file pone.0257026.s008.docx]

**Cation Complexation by Mucoid *Pseudomonas aeruginosa* Extracellular Polysaccharide**

Oliver J. Hills, James Smith, Andrew Scott, Deirdre A. Devine & Helen F. Chappell

**Supplementary information**

Mulliken bond populations and bond lengths for all Mg^2+^-oxygen contacts in each polyuronate-ion complex is given in Tables 5. For each contact, the oxygen functional group is indicated.

**Table 5**: Bond populations and lengths for the Mg^2+^-oxygen contacts in the magnesium 2-chain complexes.

| Magnesium PolyM_(ap)_ complex | | |
| --- | --- | --- |
| Bond | Population (\|e\|) | Length (Å) |
| Mg1-O1 (Ring O)  Mg1-O4 (OH)  Mg1-O7 (COO^-^)  Mg1-O50 (COO^-^)  Mg1-O51 (COO^-^)  Mg2-O9 (OH)  Mg2-O46 (Glycosidic O)  Mg2-O50 (COO^-^)  Mg2-O54 (Acetyl)  Mg3-O12 (Ring O)  Mg3-O16 (COO^-^)  Mg3-O21 (OH)  Mg-O44 (COO^-^)  Mg3-O45 (COO^-^)  Mg4-O19 (Glycosidic O)  Mg4-O24 (COO^-^)  Mg4-O27 (Acetyl)  Mg4-O33 (COO^-^) | 0.13  0.16  0.19  0.06  0.02  0.15  0.21  0.17  0.18  0.12  0.18  0.1  0.09  0.07  0.13  0.21  0.12  0.17 | 2.08  2.14  1.92  2.14  2.1  2.04  2.09  1.94  1.93  2.12  1.94  2.06  2.04  2.08  2.21  1.91  1.96  1.91 |
| Magnesium PolyMG_(p)_ complex | | |
| Bond | Population (\|e\|) | Length (Å) |
| Mg1-O10 (COO^-^)  Mg1-O11 (COO^-^)  Mg1-O27 (Ring O)  Mg1-O37 (COO^-^)  Mg2-O7 (OH)  Mg2-O8 (OH)  Mg2-O9 (Glycosidic O)  Mg2-O22 (COO^-^)  Mg2-O49 (COO^-^)  Mg3-O18 (Ring O)  Mg3-O24 (COO^-^)  Mg3-O40 (Ring O)  Mg3-O42 (OH)  Mg3-O43 (Glycosidic O)  Mg4-O13 (COO^-^)  Mg4-O30 (OH)  Mg4-O31 (Glycosidic O)  Mg4-O32 (Ring O)  Mg4-O38 (COO^-^) | 0.07  0.09  0.13  0.16  0.13  0.07  0.17  0.21  0.15  0.19  0.22  0.11  0.16  0.15  0.22  0.13  0.15  0.14  0.21 | 2.02  2  2.05  1.91  2.1  2.17  2.16  1.94  1.96  2.12  1.89  2.26  2.04  2.12  1.87  2.15  2.32  2.21  1.96 |
